# Prevalence and predictors for compensated Advanced Chronic Liver Disease (c-ACLD) in patients with chronic Hepatitis Delta Virus (HDV) infection

**DOI:** 10.1371/journal.pone.0174453

**Published:** 2017-03-22

**Authors:** Ingrid Couto, Marilu Victoria, Valdiléa G. Veloso, Lorena Rodrigues, Beatriz Grinsztejn, Marcus Lacerda, Flamir Victoria, Hugo Perazzo

**Affiliations:** 1 Fundação de Medicina Tropical Dr. Heitor Vieira Dourado (FMT HVD), Manaus, Amazonas, Brazil; 2 Laboratório de Pesquisa Clínica em DST/AIDS (LAPCLIN-AIDS), Instituto Nacional de Infectologia Evandro Chagas - Fundação Oswaldo Cruz (FIOCRUZ), Rio de Janeiro, Brazil; 3 Diretoria de Ensino e Pesquisa, Fundação de Medicina Tropical Dr. Heitor Vieira Dourado (FMT HVD), Manaus, Brazil; 4 Instituto de Pesquisas Leônidas & Maria Deane - Fundação Oswaldo Cruz (FIOCRUZ), Manaus, Brazil; Kaohsiung Medical University Chung Ho Memorial Hospital, TAIWAN

## Abstract

**Objective:**

The study aimed to evaluate the prevalence and predictor factors for compensated advanced chronic liver disease (c-ACLD) in patients with hepatitis Delta virus (HDV) infection.

**Methods:**

This cross-sectional study included consecutive HDV-infected patients defined by positive anti-HDV. Patients with hepatitis C coinfection, liver transplantation or presence of conditions that limit liver (LSM) or spleen stiffness measurement (SSM) were excluded. Blood tests, abdominal ultrasound, SSM and LSM by transient elastography (FibroScan^®^) were performed at the same day. Alcohol consumption was quantified using the AUDIT score and c-ACLD was defined by LSM ≥ 15 kPa performed by an experimented operator blinded for clinical and laboratory data.

**Results:**

101 patients were eligible and few patients were excluded due to negative anti-HDV (n = 7), hepatitis C coinfection (n = 2), liver transplantation (n = 10) and limitation for LSM or SSM (n = 5). Therefore, 77 patients [61% male, age = 43 (IQR,36–52) years] were included. The prevalence of c-ACLD was 57% (n = 44/77). Patients with c-ACLD had a higher rate of detectable HBV viral load (p = 0.039), higher levels of transaminases, GGT, alkaline phosphatases, total bilirubin and INR (p<0.001 for all), as well as lower platelet count and albumin levels (p>0.001 for both) compared to those without c-ACLD. Patients with c-ACLD had higher SSM [65.2 (IQR,33.8–75.0) vs 21.8 (16.5–32.0) kPa; p<0.001] and higher splenic volume [475 (IQR,311–746) vs 154 (112–283) cm^3^; p<0.001] compared to those without. Detectable HBV viral load (>10 UI/ml), alkaline phosphatase (per IU/L) and GGT levels (per IU/L) were independently associated with c-ACLD in all multivariate models. Splenic volume [per cm^3^,OR = 1.01 (95%CI,1.01–1.02);p = 0.002], SSM [per kPa, OR = 1.04 (1.01–1.07);p = 0.012] and splenomegaly [yes vs no,OR = 28.45 (4.42–182.95);p<0.001] were independently associated with c-ACLD.

**Conclusions:**

The prevalence of c-ACLD was high in patients with chronic HDV infection in western Amazon basin. HBV viral load, liver enzymes and splenic features can be used to predict severe liver disease in HDV-infected patients.

## Introduction

Hepatitis Delta virus (HDV) requires the hepatitis B surface antigen (HBsAg) for replication and this coinfection has been associated with severe liver disease [1]. HDV infection is endemic in Mediterranean countries, the Middle East, Central Africa, and northern parts of South America, such as the western Amazon basin. Up to 15–20 million of individuals are estimated to be infected with HDV worldwide [2]. In addition, its incidence has been increasing in nothern and central Europe mainly due to immigration from endemic regions [3]. Studies have been showing that chronic HDV infection leads to more severe liver disease than chronic hepatitis B monoinfection [4]. In addition, HDV has been associated with an accelerated course of fibrosis progression and increased risk of hepatocellular compared to other types of viral hepatitis [5].

In chronic liver diseases, the determination of liver fibrosis stage has important implications for prognostic, therapeutic and monitoring purposes. Hepatocellular carcinoma surveillance and screening of oesophageal varices should be performed in presence of cirrhosis and liver transplantation should be indicated in end-stage liver disease [6]. Historically, liver biopsy has been used to stage liver fibrosis. However, this method has been challenged by limited feasibility, potential complications, sampling error and interobserver variability [7]. Transient elastography (TE) stages fibrosis based on liver stiffness measurement (LSM) [8] and has been recommended for liver fibrosis assessment in chronic viral hepatitis [9]. The use of TE in clinical practice has allowing the early identification of patients at risk of liver-related complications. For these patients, the alternative term “compensated advanced chronic liver disease” (c-ACLD) was proposed by the Baveno VI consensus to better reflect the spectrum of severe fibrosis and cirrhosis [10]. Spleen stiffness measurement (SSM) by TE and presence of increased spleen volume have been described as surrogate markers of severity of liver disease [11]. However, few studies have assessed liver fibrosis in patients with chronic HDV infection using non-invasive methods and its correlation with SSM. The primary aim of this study was to evaluate the prevalence and predictor factors for presence of c-ACLD in patients with chronic HDV infection using TE.

## Material and methods

### Study design

This cross-sectional study was conducted on the *Fundação de Medicina Tropical Heitor Vieira Dourado* (FMT-HVD), a reference center for treatment of tropical diseases in Manaus (Amazonas), Brazil. Patients followed in the Liver Unit of this center were invited by telephone from January to March 2016 to participate and they received text messages 48h and 24h before medical appointment for protocol procedures. Patients older than 18 years with chronic infection by HDV, characterized by positive anti-HDV, were included. The exclusion criteria were hepatitis C coinfection, liver transplantation or presence of conditions that limit or contra-indicate the performance of liver or spleen stiffness, such as splenectomy, right lobe hepatectomy, ascites, pacemaker or pregnancy. Patients enrolled were submitted to the following procedures at the same day: (i) clinical evaluation; (ii) blood tests; (iii) abdominal ultrasound (US); (iv) spleen and liver stiffness measurement. The study protocol was conducted in accordance with the Helsinki Declaration, and was approved by the Ethics Committee from Fundação de Medicina Tropical Heitor Vieira Dourado (IRB n° 46865015.8.1001.0005). All patients signed an informed consent upon enrollment in the study.

### Clinical evaluation and blood tests

Clinical records included the measures of body mass index (BMI), waist circumference, blood pressure, and the reports on alcohol consumption (quantified using the Alcohol Use Disorders Identification Test [AUDIT]) and smoking (quantified in pack-years). Presence of alcohol intake was defined whether the patient had at least 1 point in the first question of AUDIT ("How often do you have a drink containing alcohol?). Patients were categorized as hazardous drinkers whether they have an AUDIT score ≥ 8 points [12]. Metabolic syndrome was defined according to the International Diabetes Federation criteria (http://www.idf.org/metabolic-syndrome). Serum alanine transaminase (ALT), aspartate transaminase (AST), gamma-glutamyltransferase (GGT), alkaline phosphatase and total bilirubin were measured by a Konelab 600i Analyzer (Thermo Electron Corporation, Vantaa, Finland). Prothrombin time and international normalized ratio (INR) were measured by a compact 1-channel coagulation instrument HumaClot Junior (Human Diagnostics Worldwide, Wiesbaden, Germany). Plasma hepatitis B viral (HBV) load was measured using Abbott RealTime HBV assays by polymerase chain reaction (PCR) using the Abbott m2000 System (Abbott, Illinois, USA).

### Abdominal ultrasound

All patients underwent abdominal ultrasound (US) (TITAN Ultrasound System, probe C60/5-2 MHz, FujiFilm SonoSite, Bothell, Washington, USA) performed by a single operator (FV) who was blinded to clinical data and blood tests results or liver and spleen stiffness measurements. Abdominal US was used for measurement of spleen diameters and to identify the best access to the spleen for spleen stiffness. Splenic maximum width (W), thickness (T) and length (L) were assessed in cm and the splenic volume was estimated according as previously described (0.524 x W x T x L) [13]. Splenomegaly was defined in presence of a spleen diameter ≥ 12 cm.

### Liver and spleen stiffness measurement

LSM was performed by a single experienced operator (HP), blinded to clinical data and blood tests results, following a validated procedure using an M probe of TE by FibroScan (EchoSens, Paris, France) [8, 14]. Patient was placed in dorsal decubitus with right arm in maximal abduction and probe placed perpendicularly in an intercostal space at the level of the right lobe of the liver. LSM was considered as the median of all valid measurements and was expressed in Kilopascal (kPa). LSM was considered reliable when the following criteria had been met: (i) 10 successful measurements; (ii) an interquartile range (IQR) lower than 30% of the median value; and (iii) a success rate of more than 60% [15]. Presence of compensated advanced chronic liver disease (c-ACLD) was defined when LSM ≥ 15 kPa [10].

SSM was performed by a single experienced operator (HP), blinded to clinical data and blood tests results using a M or XL probe of TE by FibroScan (EchoSens, Paris, France). SSM was performed in a previously US targeted point with spleen parenchyma. Briefly, the probe was placed in a left-hand side intercostal space in a dorsal decubitus patient with left arm in maximal abduction. SSM was considered as the median of all valid measurements, expressed in kPa and the examination was reliable in presence of 10 successful measurements. The XL probe was used in patients with unreliable SSM.

### Serological biomarkers of liver fibrosis

The upper limit of normal (ULN) for ALT and AST were 44 IU/L and 38 IU/L, respectively. Aspartate-to-Platelet Ratio Index (APRI) and Fibrosis-4 score (FIB-4) were calculated according the following formulas: (i) APRI = [AST(/ULN)/platelet count]*100; (ii) FIB-4 = [(age*AST)/(platelet count*[sqrt(ALT)])]. Advanced fibrosis were defined by APRI ≥1.5 [16] or FIB-4 ≥ 3.25 [17].

### Statistical analysis

Continuous variables were reported as median (IQR). Discrete variables were reported as absolute (n) and relative frequency (%). Comparisons between groups were assessed by non-parametric tests: (i) Mann-Whitney for quantitative comparisons and (ii) X^2^ test for qualitative comparisons. Variables found be associated with the presence of c-ACLD (p value < 0.20) were entered into multivariate backward stepwise logistic regression analysis. Splenic volume, spleen stiffness and presence of splenomegaly were separately entered into each multivariate model because of the strong correlation among these parameters. To avoid the effect of collinearity, Child-Pugh classification and metabolic syndrome were used in multivariate models instead of its clinical or laboratory parameters. In addition, presence of detectable HBV viral load (yes vs no) was entered into multivariate models instead of antiviral treatment (yes vs no) or HBV viral load (in log_10_ copies/ml). Significance level was determined when p value ≤ 0.05 assuming two-tailed tests.

Linear correlation and concordance between methods were assessed by Spearman's rank correlation coefficient (*r*) and Kappa value (*k*). Area under the receiver operating characteristic (AUROC) curve of splenic volume and SSM were used to assess its diagnostic value for identifying the presence of c-ACLD. The best cut-off values were chosen based on the higher rate of correctly classified patients. Diagnostic value of splenic volume and SSM were calculated by sensitivity, specificity and positive likelihood ratio (LR+). Statistical analyses were performed using STATA statistical package for Windows (2012; StataCorp LP, College Station, TX, USA).

## Results

**Fig 1:**
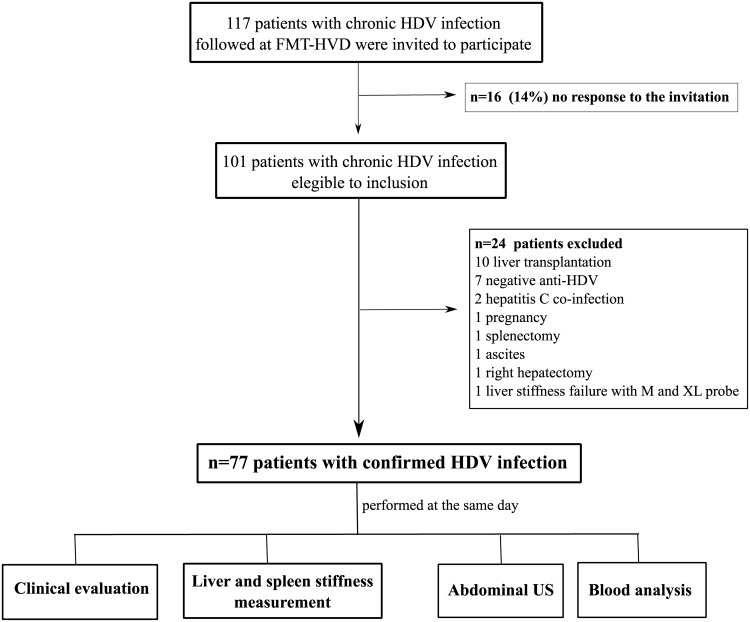
Flow-chart. Study flow chart of patient's recruitment.

A total of 117 consecutive patients with chronic HDV infection and regularly followed at FMT-HVD were invited to participate. A positive response was obtained in 86% of cases, thus 101 patients were eligible to participate. Few patients were excluded due to negative anti-HDV (n = 7), chronic hepatitis C coinfection (n = 2), liver transplantation (n = 10) and limitation or contra-indication for LSM or SSM (n = 5) (Fig 1). Therefore, 77 HDV-3 genotype patients [61% male; median (IQR) age of 43 (36–52) years and BMI of 25.9 (23.5–28.7) Kg/m^2^, 83% HBeAg negative/anti-HBe positive] were included in the analysis. Regarding alcohol consumption, 72% (n = 55) of patients had no alcohol intake (AUDIT score = 0) and only five (7%) patients were considered as hazardous drinkers (AUDIT score ≥ 8). Most patients were under treatment with nucleos(t)ide analogues (NUCs) or had previous IFN-based regimens and HBV viral load was positive (>10 UI/ml) in 27% of cases (n = 21).

**Fig 2:**
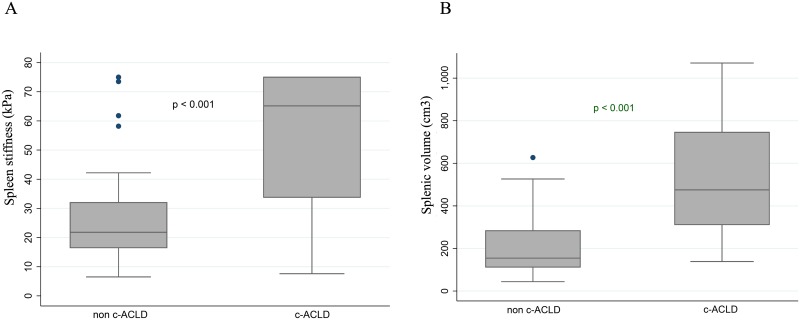
Diagnosis of c-ACLD by spleen features. Spleen stiffness measurement (A) and splenic volume (B) according to diagnosis of compensated advanced chronic liver disease (c-ACLD) by transient elastography.

The prevalence of c-ACLD according to LSM (≥ 15 kPa) was 57% (n = 44/77). The median LSM, SSM and splenic volume were 13.8 kPa (IQR 6.2–31.2), 35.8 kPa (21.1–75.0) and 348 cm^3^ (156–560), respectively. Patients with c-ACLD had a higher rate of detectable HBV viral load (p = 0.039), higher levels of transaminases, GGT, alkaline phosphatases, total bilirubin and INR (p<0.001 for all), as well as lower platelet count and albumin levels (p>0.001 for both) compared to those without c-ACLD. Patients with detectable HBV-DNA had significantly higher median (IQR) LSM [19.0 (12.0–39.7) vs 11.5 (5.8–25.2) kPa; p = 0.013] compared to those with undetectable HBV-DNA. In addition, c-ACLD patients had significantly higher SSM [65.2 (IQR, 33.8–75.0) vs 21.8 (16.5–32.0) kPa; p<0.001] and higher splenic volume [475 (IQR, 311–746) vs 154 (112–283) cm^3^; p<0.001] compared to those without c-ACLD (Fig 2). Serological biomarkers were associated with c-ACLD: (i) APRI ≥ 1.5 [OR = 41.33 (95%CI 8.55–199.94) and k values = 0.64 (p < 0.001 for both)]; (ii) FIB-4 ≥ 3.25 [OR = 26.67 (6.85–103.86) and k values = 0.64 (p < 0.001 for both)]. In addition, LSM was highly correlated with APRI and FIB-4 values: Spearman’s value (*r*) of 0.743 and 0.738 (p < 0.001 for both), respectively. Table 1 describes the characteristics of patients according to presence or absence of c-ACLD based on LSM.

**Table 1:** Clinical and demographic characteristics of patients with chronic hepatitis Delta infection according to severity of liver disease by transient elastography.

	All (n = 77)	non-cACLD (n = 33)	cACLD(n = 44)	p value
Male gender ^a^	47 (61)	19 (58)	28 (64)	0.589
Age, years ^b^	43 (23–72)	44 (23–71)	43 (23–72)	0.585
Metabolic syndrome ^a^	7 (9)	4 (12)	3 (7)	0.423
Alcohol use ^a^	8 (10)	3 (9)	5 (11)	0.746
AUDIT score ^c^	0 (0–2)	0 (0–0)	0 (0–2)	0.511
Tabaco use ^a^	42 (55)	17 (52)	25 (57)	0.644
Packs-years smoking ^c^	5 (1–15)	7 (2–15)	3 (1–18)	0.578
**HBV status**				
Antiviral treatment (yes vs no) ^a^				0.135
No treated vs type of treatment				0.042
No treatment	15 (20)	9 (27)	6 (14)	
Interferon-based treatment	19 (25)	11 (33)	8 (18)	
NUC-based treatment	43 (55)	13 (40)	30 (68)	
HBV viral load, log ^c^	0.00 (0.00–1.15)	0.00 (0.00–0.00)	0.00 (0.00–1.28)	0.057
Detectable HBV viral load, > 10 UI/ml ^a^	21 (27)	5 (15)	16 (36)	0.039
**Spleen status**				
Spleen volume, cm3 ^c^	348 (156–560)	154 (112–283)	475 (311–746)	<0.001
Splenomegaly, diameter > 12cm ^a^	36 (47)	4 (12)	32 (73)	<0.001
Spleen stiffness, kPa ^b^	35.8 (21.1–75.0)	21.8 (16.5–32.0)	65.2 (33.8–75.0)	<0.001
**Liver function**				
ALT, IU/L ^b^	38 (8–172)	26 (8–136)	55 (12–172)	<0.001
AST, IU/L ^b^	42 (16–154)	28 (16–118)	65 (16–154)	<0.001
Alkaline Phosphatases, IU/L ^b^	233 (39–572)	199 (119–289)	283 (39–572)	<0.001
GGT, IU/L ^b^	35 (11–254)	21 (11–84)	79 (15–254)	<0.001
Total bilirubin, mg/dL ^b^	1.07 (0.04–5.59)	0.66 (0.04–3.00)	1.32 (0.45–5.59)	<0.001
Albumin, g/dL ^b^	4.6 (3.2–6.4)	4.9 (4.1–6.4)	4.3 (3.2–5.5)	<0.001
INR ^b^	1.2 (1.0–2.1)	1.1 (1.0–1.3)	1.3 (1.0–2.1)	<0.001
Platelet count, x 103 / mm3 ^b^	109 (20–294)	155 (41–294)	54 (20–250)	<0.001
Child-Pugh score ^c^	5 (5–6)	5 (5–5)	6 (5–7)	0.003
Child-Pugh B/C ^a^	13 (17)	2 (6)	11 (25)	0.028
**Serological biomarker of fibrosis**				
APRI ^c^	0.98 (0.45–3.78)	0.46 (0.34–0.67)	2.80 (1.35–5.68)	<0.001
APRI ≥1.5	34 (44)	2 (6)	32 (73)	<0.001
FIB-4 ^c^	2.97 (1.52–8.27)	1.61 (1.14–2.57)	7.79 (2.84–11.37)	<0.001
FIB-4 ≥ 3.25	35 (46)	3 (9)	32 (72)	<0.001

Data expressed as (a) absolute (%),(b) median (min-max) or (c) median (interquartile range). Types of treatment (n = 62): interferon monotherapy (n = 12); peginterferon monotherapy (n = 7); entecavir monotherapy (n = 41); combined lamivudine-tenovofir (n = 2).ALT, alanine transaminase; APRI, Aspartate-to-Platelet Ratio Index; AST, aspartate transaminase; c-ACLD, compensated advanced chronic liver disease; FIB-4, Fibrosis-4 score; GGT, gamma-glutamyltransferase; HBV, hepatitis B virus; INR, international normalized ratio. c-ACLD was defined as transient elastography ≥ 15 kPa.

SSM by the M probe was considered as unreliable in 16.9% (n = 13) of cases, thus spleen stiffness was measured using the XL probe in these patients. The following factors were significantly associated with unreliability of spleen stiffness with M probe [OR (95%CI), p value]: female gender [4.61 (1.27–16.71), p = 0.020], presence of metabolic syndrome [9.04 (1.73–47.17), p = 0.009], absence of antiviral treatment [8.17 (2.19–30.52), p = 0.002] and absence of splenomegaly [6.23 (1.28–30.40), p = 0.024], as well as normal spleen volume [6.75 (1.87–24.93), p = 0.004] and platelet count > 150 x 10^9^/mm^3^ [13.08 (3.14–54.46), p<0.001] (Table 2).

**Table 2:** Risk factors associated with unreliability of spleen stiffness by the M probe (SSM-M).

	Reliable SSM-M (n = 64)	Unreliable SSM-M (n = 13)	OR (95%CI)	p value
Female gender ^a^	21 (33)	9 (69)	4.61 (1.27–16.71)	0.020
Age, years ^b^	43 (36–51)	46 (39–52)	1.03 (0.98–1.09)	0.207
Metabolic syndrome ^a^	3 (5)	4 (31)	9.04 (1.73–47.17)	0.009
Alcohol use ^a^	6 (9)	2 (15)	1.76 (0.31–9.87)	0.522
Tabac use ^a^	34 (53)	8 (62)	1.41 (0.42–4.78)	0.580
**HBV status**				
No antiviral treatment ^a^	8 (12)	7 (54)	8.17 (2.19–30.52)	0.002
HBV viral load, log ^b^	0.00 (0.00–1.15)	0.00 (0.00–1.84)	0.86 (0.42–1.75)	0.673
Detectable HBV viral load, > 10 UI/ml ^b^	18 (28)	3 (23)	0.77 (0.19–3.11)	0.710
**Spleen status**				
Normal spleen volume, < 192 cm3 ^a^	16 (25)	9 (69)	6.75 (1.87–24.93)	0.004
Absence of splenomegaly,diameter ≤ 12cm ^a^	30 (47)	11 (85)	6.23 (1.28–30.40)	0.024
**Liver function**				
ALT, IU/L ^b^	43 (28–69)	22 (17–39)	0.98 (0.95–1.01)	0.087
AST, IU/L ^b^	44 (29–76)	26 (20–41)	0.97 (0.94–1.00)	0.060
Alkaline Phosphatases, IU/L ^b^	243 (199–338)	167 (145–234)	0.98 (0.97–0.99)	0.015
GGT, IU/L ^b^	53 (21–87)	25 (17–29)	0.96 (0.93–0.99)	0.026
Total bilirubin, mg/dL ^b^	1.19 (0.72–1.88)	0.54 (0.42–0.75)	0.04 (0.01–0.36)	0.004
Albumin, g/dL ^b^	4.6 (4.0–4.9)	4.8 (4.6–4.9)	2.09 (0.71–6.15)	0.181
Normal platelet count, ≥ 150 x 10^9^ / mm^3a^	13 (20)	10 (77)	13.08 (3.14–54.46)	<0.001
Child-Pugh B/C ^a^	12 (19)	1 (8)	0.36 (0.04–3.05)	0.350

Data expressed as (a) absolute (%) or (b) median (interquartile range). ALT, alanine transaminase; AST, aspartate transaminase; GGT, gamma-glutamyltransferase; HBV, hepatitis B virus; OR, odds ratio; CI, confidence interval

**Fig 3:**
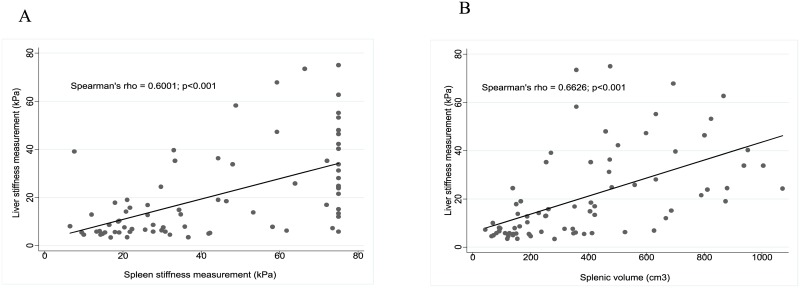
Linear correlation between liver and spleen features. Linear correlation (Sperman's rho, p value) between spleen and liver stiffness measurement (A) and between splenic volume and liver stiffness measurement (B).

SSM and splenic volume were correlated with LSM: Spearman’s value (*r*) of 0.600 (p < 0.001) and 0.663 (p < 0.001), respectively (Fig 3). Detectable HBV viral load (yes vs no), platelet count < 150 x 10^9^/mm^3^ (yes vs no), presence of Child-Pugh B/C (yes vs no) and levels (per UI/L) of ALT, AST, GGT and alkaline phosphatase were included in the multivariate models. Presence of detectable HBV viral load [(yes vs no) and levels (per UI/L) of GGT and phosphatase alkaline were independently associated with c-ACLD in all multivariate models (Table 3). In addition, splenic volume [per cm^3^, OR = 1.01 (95%CI 1.01–1.02); p = 0.002], SSM [per kPa, OR = 1.04 (1.01–1.07); p = 0.012] and presence of splenomegaly [yes vs no, OR = 28.45 (4.42–182.95); p<0.001] were independently associated with c-ACLD when entered in distinct models (Table 3).

**Table 3:** Univariate and multivariate analysis for factors associated with compensated Advanced Chronic Liver Disease (c-ACLD) based on liver stiffness measurement by transient elastography.

	Univariate analysis		Multivariate analysis					
			Model I		Model II		Model III	
	OR	p	OR	p	OR	p	OR	p
	[95% CI]	value	[95% CI]	value	[95% CI]	value	[95% CI]	value
Gender (male vs. female)								
	1.30	0.590						
	[0.51–3.25]							
Age (per year increase)								
	0.99	0.614						
	[0.95–1.03]							
Metabolic syndrome (yes vs. no)								
	0.53	0.429						
	[0.11–2.55]							
Alcohol use (yes vs. no)								
	1.28	0.747						
	[0.28–5.79]							
Tabac use (yes vs. no)								
	1.24	0.644						
	[0.50–3.07]							
Antiviral treatment (yes vs. no)								
	2.38	0.141						
	[0.75–7.52]							
HBV viral load (per log)								
	1.67	0.082						
	[0.94–2.99]							
Detectable HBV viral load (yes vs. no)								
	3.20	0.044	6.04	0.027	10.86	0.023	7.25	0.043
	[1.03–9.93]		[1.22–29.81]		[1.38–85.37]		[1.07–49.32]	
Spleen stiffness (per kPa)								
	1.06	<0.001	1.04	0.012				
	[1.03–1.09]		[1.01–1.07]					
Spleen volume (per cm3)								
	1.01	<0.001			1.01	0.002		
	[1.01–1.02]				[1.01–1.02]			
Splenomegaly (yes vs. no)								
	19.33	<0.001					28.45	<0.001
	[5.61–66.68]						[4.42–182.95]	
ALT (per IU/L)								
	1.05	<0.001						
	[1.02–1.07]							
AST (per IU/L)								
	1.06	<0.001						
	[1.03–1.10]							
Alkaline Phosphatase (per IU/L)								
	1.02	<0.001	1.01	0.041	1.01	0.022	1.02	0.025
	[1.01–1.03]		[1.01–1.02]		[1.01–1.03]		[1.01–1.03]	
GGT (per IU/L)								
	1.05	<0.001	1.04	0.010	1.04	0.005	1.05	0.002
	[1.03–1.08]		[1.01–1.07]		[1.01–1.08]		[1.02–1.08]	
Platelet count < 150 x 109/mm^3^ (yes vs. no)								
	6.73	0.001						
	[2.42–20.19]							
Child-Pugh B/C (yes vs. no)								
	5.17	0.042						
	[1.06–25.19]							

Multivariate backward stepwise logistic regression analysis: detectable HBV viral load, liver function tests, platelet count < 150 x 10^9^/mm^3^ and child-pugh class entered in all multivariate models. Spleen status entered in separate models: spleen stiffness (kPa) in Model I; spleen volume (cm3) in Model II and splenomegaly (spleen diameter ≥ 12cm) in Model III

**Fig 4:**
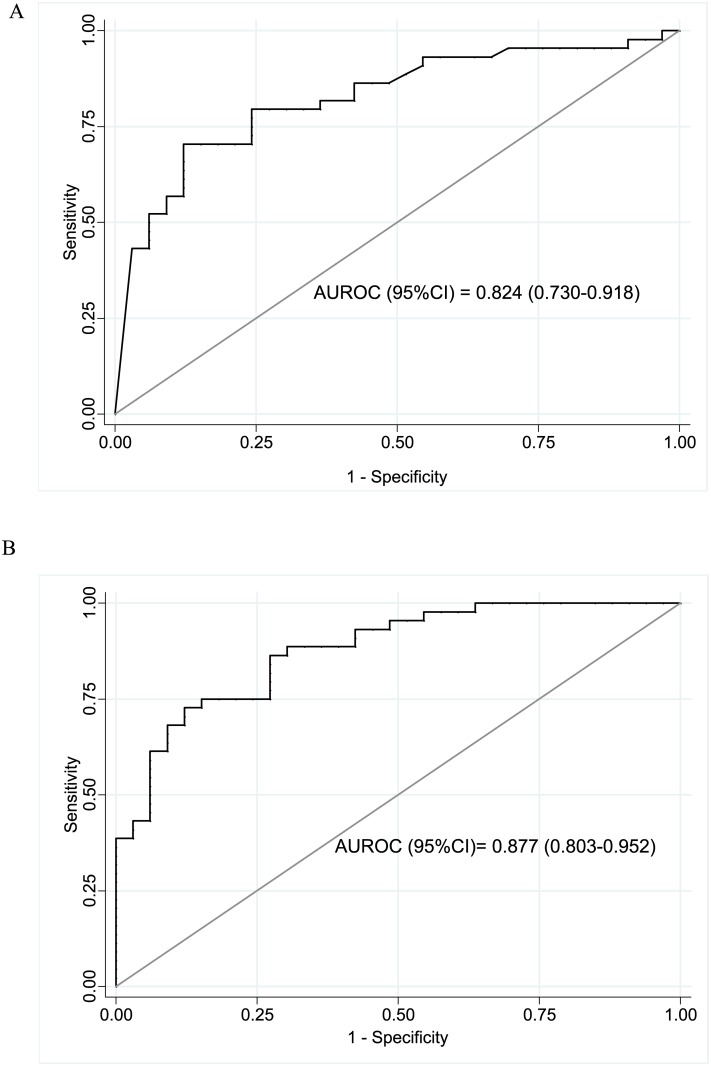
Diagnostic accuracy of spleen stiffness measurement and splenic volume. Area under the receiver operating characteristic (AUROC) curve for spleen stiffness measurement (A) and splenic volume (B) for prediction of compensated advanced chronic liver disease (c-ACLD) by transient elastography.

The AUROC (95%CI) of SSM and splenic volume to predict c-ACLD were 0.824 (0.730–0.918) and 0.877 (0.803–0.952), respectively (Fig 4). Regarding the diagnostic performance of SSM to predict c-ACLD, the optimal identified cut-off was 44.3 kPa that correctly classified 77.9% of patients and yielded a sensitivity (95% CI), specificity (95% CI) and LR+ of 66% (52–80), 88% (77–99) and 5.5, respectively. In addition, the optimal cut-off identified for splenic volume was 252 cm^3^ that correctly classified 79.2% and yielded a sensitivity of 84% (73–95), specificity of 73% (58–88) and LR+ of 3.1.

## Discussion

This study highlighted the high prevalence of c-ACLD (57%) using LSM by TE to stage liver fibrosis in patients with chronic HDV infection. HBV viral load, alkaline phosphatase, GGT levels, spleen stiffness (in kPa), spleen volume (in cm^3^) and presence of splenomegaly were independently associated with c-ACLD in this population of patients from northern Brazil.

Braga et al reported a prevalence of 33% for advanced fibrosis (METAVIR F≥3) in genotype-3 HDV infected patients staged by liver biopsy in western Amazon basin [18]. This prevalence was up to 40% in a European study that included genotype-1 HDV patients [19] and up to 53% in multi-center study in Italy [1]. The present study reported a higher prevalence of severe liver disease in chronic HDV infection than those series. The conflicting results between the Brazilian studies might be explained by the fact that we included older patients [median (min-max) age of 43 (23–72) vs 25 (17–55) years] with lower platelet count [109 (20–294) vs 155 (55–468) x 10^9^/mm^3^]. We probably included more severe patients than those studies because 17% of our population were Child-Pugh B/C and most of our patients (80%) was under treatment by NUCs or had previous IFN-based regimens. The European patients were recruited for a multicenter randomized clinical trial where antiviral therapy for chronic HDV within the previous 6 months, platelet count < 90 x 10^9^/mm^3^ and Child-Pugh B/C were exclusion criteria. The low prevalence of abusive alcohol intake (7%) and metabolic syndrome (9%) in the present study might explain the low statistical significance with c-ACLD. Similar results were observed when replacing metabolic syndrome by its individual features and abusive by any alcohol intake.

Calle Serrano et al described that older age, male gender, Eastern Mediterranean origin, higher levels of bilirubin and coagulation disturbances were associated with higher rates of liver-related morbidity or mortality in hepatitis delta [20]. In addition, anti-HDV IgM might be used as a serological biomarker for disease activity in HDV infection [21]. Few studies have evaluated the role of splenic and hepatic elastography for prediction of portal hypertension or advanced fibrosis in chronic viral hepatitis. SSM by TE ranging from 40 to 60 kPa has been described as optimal for prediction of clinical significant portal hypertension defined by hepatic venous pressure gradient (HVPG) in chronic hepatitis C [22, 23]. Wong et al reported that SSM = 21.4 kPa could be used for rule-out [sensitivity = 90.3% (95%CI 75.1–96.7)] and SSM = 50.5 kPa for rule-in [specificity = 91.3% (95%CI 85.0–95.1)] esophageal varices in patients with chronic hepatitis B [24]. However, this study included a limited sample size (n = 144) and few patients (25%) had esophageal varices. In another limited sample size study that evaluated patients with chronic hepatitis B (19%) or C (81%) by liver biopsy, Fraquelli et al reported that a 46 kPa SSM cut-off yielded a sensitivity (95%CI), specificity (95%CI) and AUROC (95%CI) of 89% (72–99), 78% (82–96) and 0.84 (0.76–0.93) for prediction of cirrhosis (METAVIR F = 4), respectively [11]. Our study described similar results with a 44.3 kPa SSM cut-off to predict severe liver disease, defined as c-ACLD, in patients with HDV infection, despite the fact that we used hepatic elastography as the reference.

The measurements of spleen stiffness seem to be more complicate than those performed in the liver. SSM needs a previous US to target the point of measurement, the failure rate might be up to 29% of cases and its learning curve was not evaluated. Studies have described metabolic features and absence of splenomegaly as factors associated with failure of SSM [25]. Our study revealed comparable results with presence of metabolic syndrome [9.04 (1.73–47.17), p = 0.009], normal spleen volume [6.75 (1.87–24.93), p = 0.004], absence of splenomegaly [6.23 (1.28–30.40), p = 0.024] and female gender [OR = 4.61 (95%CI 1.27–16.71), p = 0.020], as the main factors associated with unreliable SSM with the M probe (13% of cases). However, in our study, all unreliable examinations with M probe were reliable using the XL probe.

The main limitations of our study were the lack of HDV-RNA quantification and the lack of liver biopsy, as the gold standard for correctly staging liver fibrosis or surrogate markers for detection of portal hypertension. Several in-house and commercial assays for quantify HDV-RNA have been developed worldwide, but they performed poorly mainly due to HDV genetic variability and lack of standardization [26]. The present study was performed in a low-income country were HDV-RNA quantification assay and HPVG measurement were not available. We estimate that the performance of liver biopsy would not be feasible due to thrombocytopenia and ethical issues. Our population had a platelet count lower than 50 x 10^9^/mm^3^ in 27% (n = 21) of patients and ranging between 51–100 x 10^9^/mm^3^ in 18% (n = 14) of cases. In addition, peginterferon, limited by side effects andlow response rate, remains the current standard of care for treatment for HDV infection. In the HIDIT-1 study, Peginterferon-based therapy improved biochemical disease activity without decrease in liver-related complications in 5 years of follow-up [27]. It could be unethical to perform liver biopsy in patients with a coagulation disorder to indicate a treatment with low response rate. APRI and FIB-4 were strongly correlated with c-ACLD, which reinforced the presence of severe liver disease. However, these serological biomarkers use platelet count in theirs formulas and most patients with chronic HDV infection have splenomegaly and hypersplenism leading to thrombocytopenia that might overestimate liver fibrosis by methods. In addition, those serological markers had a not satisfactory diagnostic performance (AUROC < 0.80) in HDV infected patients using liver biopsy as the reference [19]. The same authors have proposed a new biomarker, Delta Fibrosis Score, that uses levels of cholinesterase, GGT and albumin combined to age, for prediction of liver fibrosis in HDV infection. However, this test was evaluated in a limited sample of a central European cohort infected by genotype-1 HDV that was included in a clinical trial (HIDIT-2; NCT00932971). Thus, further prospective studies might be needed to be used in regions with different HDV genotypes, such as Amazon basin where HDV-3 is the most frequent genotype [28].

We acknowledge that TE might not be considered as a gold standard for liver fibrosis staging. However, it seems very difficult, even using liver biopsy, to well discriminate adjacent stages of fibrosis [29]. In addition, liver fibrosis estimation by TE might be limited by interobserver variability [30] and influenced by several factors, such as food intake, presence of necro-inflammatory activity, extrahepatic cholestasis or liver congestion [31]. However, this non-invasive method has been extensively validated for hepatitis B [32], examinations were performed by a single experienced operator (> 2000 exams) in 3-hour fasting patients and those with conditions that limit liver stiffness measurement were excluded. We are aware that validation studies for non-invasive methods are scare in HDV infection and there was no validated cut-off for TE in HDV infection. To overcome this issue, we used a liver stiffness cut-off proposed for diagnosis of c-ACLD (LSM ≥ 15 kPa) in different chronic liver diseases [10]. The definition of c-ACLD is an alternative term that has been proposed by experts to better reflect that the spectrum of severe fibrosis and cirrhosis is a continuum in asymptomatic patients.

An addition technical issue is related to the upper detection limit for stiffness measurement of TE by Fibroscan (75 kPa). Previous authors reported up to 12% of patients with chronic hepatitis C had SSM equal to 75 kPa [22]. In our study, 26% (n = 20/77) of patients had spleen stiffness located in the upper limit of detection. This issue might be covered by using other types of hepatic elastography, such as shear wave supersonic that present a higher upper limit of detection for tissue stiffness. Others criticisms of our study could be the limited sample size and the fact that patients were invited to participate that might lead to a potential selection bias. HDV is a relative rare disease and previous studies recruited similar sample size. In addition, we included patients in a tertiary center located in northern Brazil (Manaus, Amazonas), an endemic region for HDV infection. All patients (n = 117) followed for HDV infection in our center were invited by telephone call and text message reminder 48h and 24h before the appointment. Generally, patients that respond to a hospital convocation might be those more attentive about their health care. However, our high response rate (86%) has probably reduced this potential selection bias.

The originality of this study remains on the report of predictor factors for liver fibrosis in patients with chronic HDV infection recruited in the Amazon basin and the description of its correlation with spleen stiffness in Latin America. Our major strength was the assessment of clinical data, blood tests and imaging methods at the same day. Experienced operators performed TE (HP) and abdominal US (FV) blinded for clinical data and laboratory results.

## Conclusions

In conclusion, compensated advanced chronic liver disease, defined by TE, was highly prevalent in a limited sample size population with chronic HDV infection in the Amazon basin. More severe liver disease was significantly associated with higher levels of HBV viral load, phosphatase alkaline and GGT. In addition, spleen stiffness, splenic volume and presence of splenomegaly were independently associated with compensated advanced chronic liver disease in these patients. Non-invasive methods to stage liver disease should be integrated to clinical management of HDV infection to decrease the burden of hepatic events in these patients.

## Abbreviations

ALT: alanine transaminase
APRI: Aspartate-to-Platelet Ratio Index
AST: aspartate transaminase
AUDIT: Alcohol Use Disorders Identification Test
AUROC: area under the receiver operating characteristic
BMI: body mass index
c-ACLD: compensated-advanced chronic liver disease
CI: confidence interval
FIB-4: Fibrosis-4 score
GGT: gamma-glutamyltransferase
HBsAg: hepatitis B surface antigen
HBV: hepatitis B virus
HDV: hepatitis Delta virus
HVPG: hepatic venous pressure gradient
INR: international normalized ratio
IQR: interquartile range
L: length
LR+: positive likelihood ratio
LSM: liver stiffness measurement
NUCs: nucleos(t)ide analogues
OR: odds ratio
PCR: polymerase chain reaction
SSM: spleen stiffness measurement
T: thickness
TE: transient elastography
US: ultrasound
W: width
